# Mid-term results of a less-invasive locking plate fixation method for proximal humeral fractures: a prospective observational study

**DOI:** 10.1186/s12891-015-0618-y

**Published:** 2015-07-04

**Authors:** Benjamin Bockmann, Benjamin Buecking, Daniel Franz, Ralph Zettl, Steffen Ruchholtz, Juliane Mohr

**Affiliations:** Center for Orthopedics and Trauma Surgery, University Hospital Giessen and Marburg, Location Marburg, Baldingerstrasse, 35043 Marburg, Germany; Department of Trauma Surgery, Otto-von-Guericke University, Leipziger Strasse 44, 39120 Magdeburg, Germany

## Abstract

**Background:**

The optimal treatment for proximal humeral fractures remains under debate. In this article, we report the mid-term results of patients who underwent the less-invasive implantation of a polyaxial locking plate for displaced proximal humeral fractures.

**Methods:**

This study included patients who were treated with a polyaxial locking plate via an anterolateral deltoid split approach from May 2008 to December 2011. We evaluated outcome parameters after a minimum follow-up period of 2.5 years (median 4.5 years, follow-up rate 62 %) including the age- and gender-dependent Constant score, the activities of daily living score, and the visual analog scale for both pain and subjective shoulder function.

**Results:**

Of the 140 patients who underwent surgery, 114 were included in the follow-up and 71 completed the questionnaire. Fifteen patients (21 %) exhibited 2-fragment fractures, and 56 patients (79 %) exhibited 3- and 4-part fractures. The Constant score improved significantly (4.5 years: 70 ± 21, *p* < 0.001) between the first two follow-ups (6 weeks: 35 ± 14, 6 months: 56 ± 18, *p* < 0.001), and also between 6 months and 4.5 years post-surgery. At the final follow-up, the activities of daily living score had not reached pre-fracture levels (before trauma: 27 ± 5, 4.5 years: 20 ± 8, *p* < 0.001). A multivariate analysis showed that age has a more significant influence on the final outcome than fracture morphology or gender.

**Conclusion:**

Although the less-invasive surgical procedure is a feasible treatment option in proximal humeral fractures with acceptable complications and considerable improvement during the first six months, a lengthy recovery time is required. The majority of our patients did not become pain-free or reach pre-fracture activity levels.

## Background

Proximal humeral fractures represent 5 % of all fractures and 45 % of all humeral fractures. Minor trauma may cause fractures in cases of reduced bone quality, which explains why more than 76 % of all humeral fractures are observed in patients older than 60 years [1–3].

Several studies have aided in furthering the understanding of the pathology of proximal humeral fractures. Concurrently, improvements have been made in visualization technologies, i.e., live 3D imaging during surgery. Thus, less-invasive surgical procedures have the potential to minimize local soft tissue trauma, resulting in lower complication rates, reduced postoperative pain, fewer periarticular adhesions, and better joint function [4]. Perioperative risk could possibly be reduced in various musculoskeletal trauma situations and even in “central injuries” such as fractures of the anterior column of the spine, the pelvic ring, and the femur [5]. Our working group recently demonstrated that a less-invasive treatment of proximal humeral fractures achieved equivalent postoperative functional results compared with conventional techniques at the 1-year follow-up [6]. However, this study encompassed two different approaches with different degrees of invasiveness.

Data on mid-term results in displaced proximal humeral fracture are scarce. It is unknown whether complications alter the final outcome or if patients may expect further improvement after 6 months. Thus, we evaluated patients’ mid-term outcomes after internal fixation using a polyaxial locking plate and an anterolateral deltoid split approach.

## Methods

A total of 140 patients from two prospective clinical trials were analyzed. Both trials evaluated the results of minimally invasive surgery (MIS)-plating in proximal humeral fractures. The procedures were performed from May 2008 to November 2011, and only patients with a displaced proximal humeral fracture were included [5, 6]. The fractures were diagnosed by plain radiographs in the a.p. and axial views and were then classified according to the Neer classification by the surgeon in the operating suite. A CT scan was not routinely performed.

Patients who met the following criteria were initially excluded: age younger than 18 years, multiple traumas, pathological fractures, combined fractures of the upper extremity, a duration between trauma and surgery of more than 10 days, preoperative nerve or vascular lesions that persisted after closed reduction, grade II and III open fractures, fractures combined with shoulder dislocations, and isolated fractures of the greater or lesser tuberosity.

Furthermore, patients who either submitted an incomplete questionnaire or who received primary or secondary joint replacement were excluded from the mid-term analysis.

Our trials were approved by the Marburg School of Medicine ethics committee (file numbers 142-69192 and 150/09). Informed consent was obtained prior to patient inclusion.

A total of 140 patients were included. The follow-up rate was 90 % after 6 weeks, 78 % after 6 months, and 62 % after 4.5 years.

**Fig. 1:**
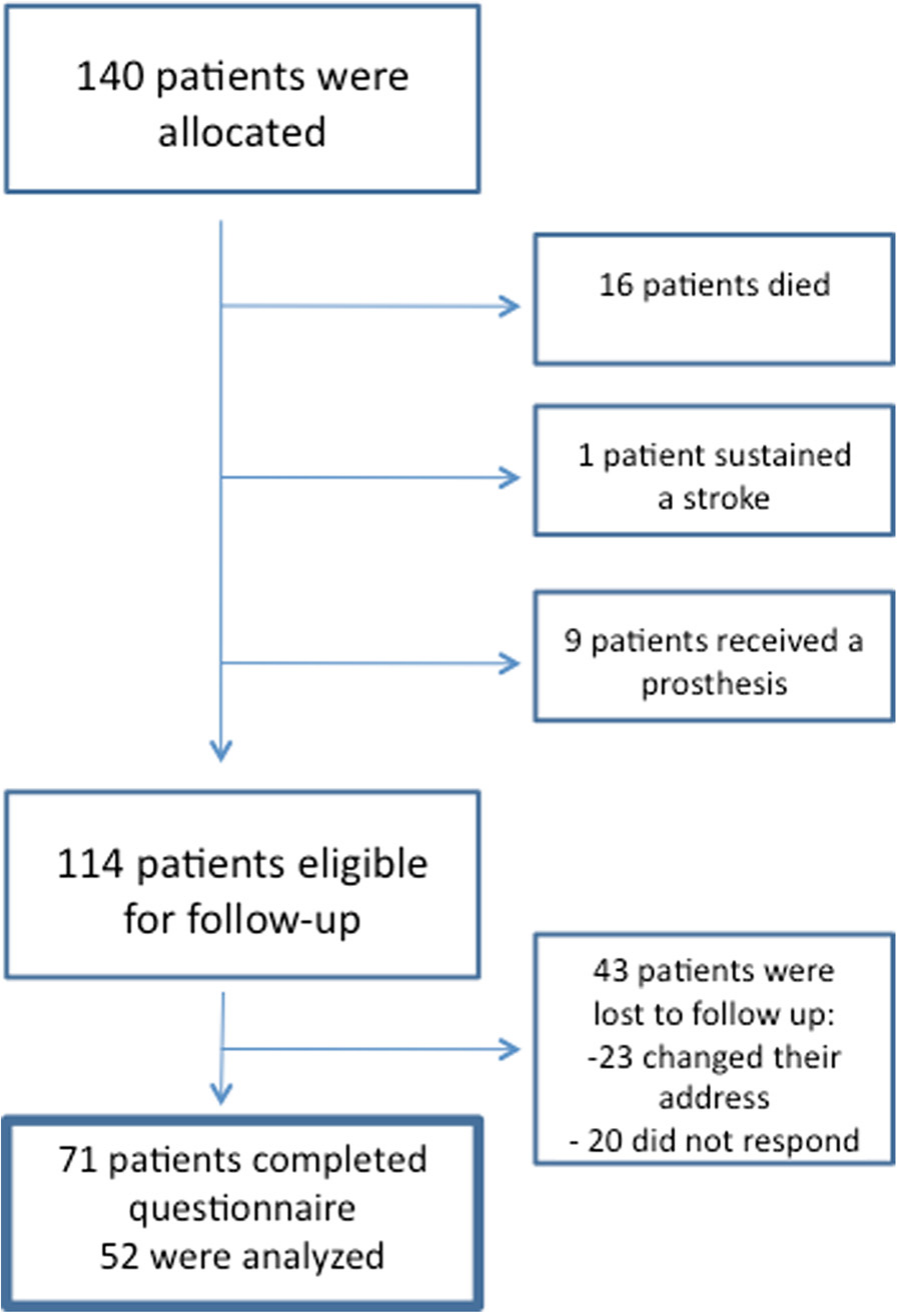
Follow-up participation

Of the 140 included patients, 16 died during follow-up. One patient had a stroke that prohibited further evaluation of shoulder function, and nine patients received a secondary prosthesis and were excluded from the follow-up. Of the 114 remaining patients, 43 could not be contacted. A total of 71 patients completed the questionnaire administered at the 4.5-year follow-up (Fig. 1). The shortest follow-up was 34 months, and the longest was 72 months.

Data from each follow-up of 52 patients were used to analyze the time course of the selected outcome parameters.

To assess potential bias, we also evaluated the data of the 43 patients who were lost to follow-up.

The procedure was conducted as previously described:

An anterolateral 3 cm deltoid split with incision of the bursa subdeltoidea was performed.

**Fig. 2:**
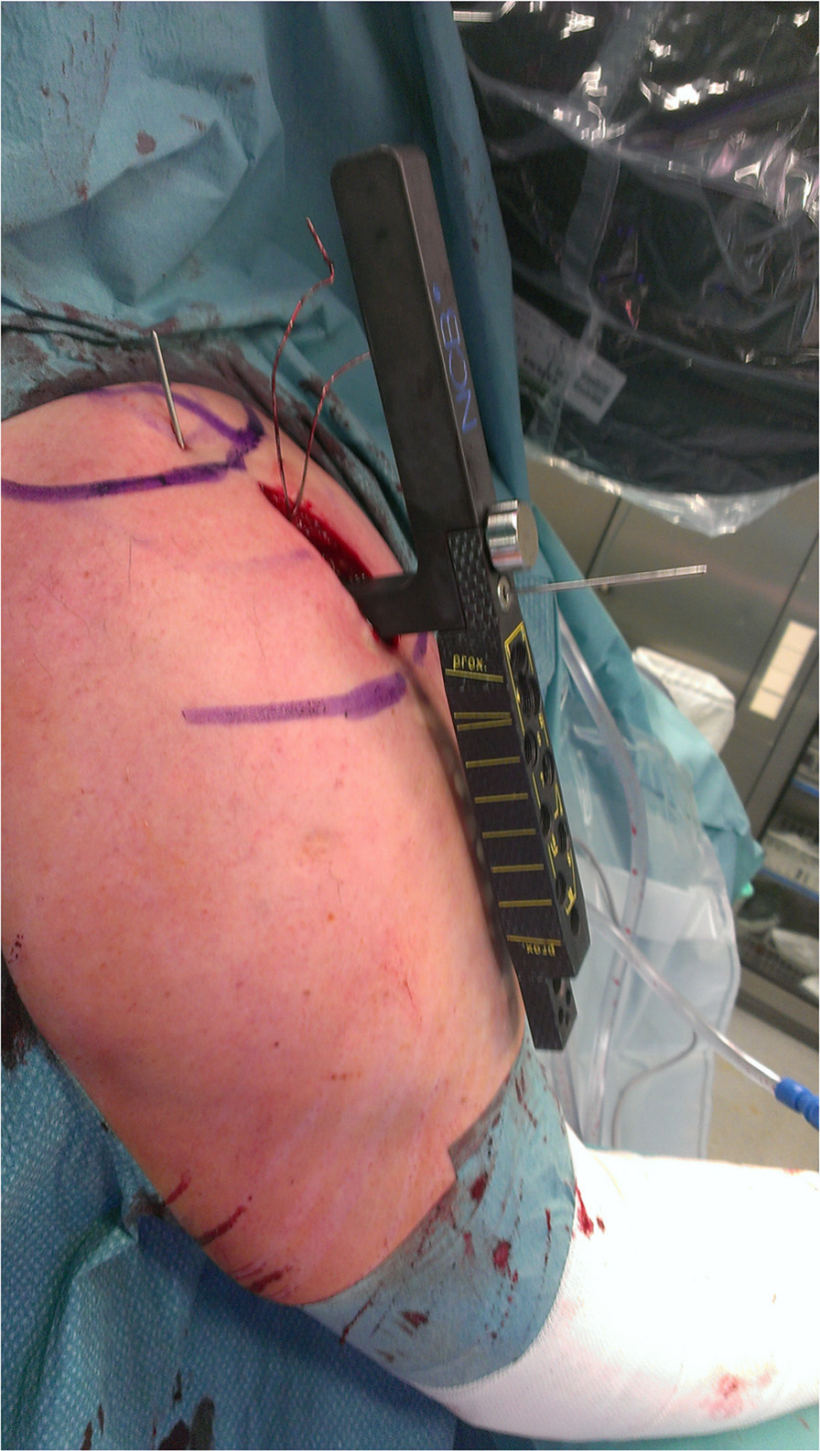
Intraoperative image of the deltoid split approach. The course of the axillary nerve is marked using a sterile pen

The axillary nerve was identified by index finger palpation through the bursa subdeltoidea, and its course was marked on the skin (Fig. 2).

A potentially displaced greater tuberosity was fixed by a stay suture or cerclage.

The fracture was reduced by ligamentotaxis (downward traction of the arm) and/or direct manipulation with pushers or rasps through the deltoid split to manipulate important fragments that were not attached to tendons or ligaments. The manipulation of pulling the arm downward simultaneously enabled the anatomic reduction. Moreover, the setting allowed the direct visualization of the manipulation by the intensifier.

The fracture was temporarily fixed by a Kirschner wire that was drilled using an oblique technique from proximal lateral (through the tip of the greater tuberosity) into the medial part of the proximal humeral shaft.

The plate was inserted below the nerve and temporarily fixed to the humeral head and the shaft by K-wires through the jig.

A long five-hole plate was percutaneously fixed with three locking screws to the diaphysis through the jig after verification of the correct position in both directions turning the forearm from neutral to a 90° internal rotation. To prevent damage to the axillary nerve, only the distal three holes were used.

The three cannulated locking screws were placed over the K-wires into the humeral head.

A displaced greater tuberosity was fixed through the holes in the plate by fiber wire or cerclage [5].

The shoulder was immobilized for the first 2 days after surgery. Early passive and limited active motion of the shoulder was initiated. Regarding abduction, however, only assisted abduction up to 90° was allowed for the first 6 weeks after surgery.

### The implant

The non-contact bridging plate (NCB-PH; Zimmer, Warsaw, IN, USA) is a polyaxial locking plate used for internal fixation of proximal humeral fractures. Using a less-invasive deltoid split approach, the plate and the shaft screws are inserted with an aiming device.

The head area consists of five holes. Additionally, there are two angular holes designated for wire cerclages and non-absorbable sutures that can be used for additional fixation of the greater tuberosity.

Previous research has shown that the biomechanical properties of the implant are feasible for daily clinical practice use. However, some authors cited the thickness of the implant as a disadvantage that could lead to secondary impingement. [4, 7]

### Assessment of mid-term results

At all follow-ups, the Constant score, which was adapted according to gender and age as described by Katolik, and the activities of daily living (ADL) score were recorded [8]. At our first follow-up, the patients were also queried to obtain their ADL scores before their injuries. Pain and subjective shoulder function were evaluated by the visual analog scale (VAS) score at discharge, 6 weeks, 6 months, and a minimum of 2.5 years (median 51 months) postoperatively. Our patients chose values on graphic scales from 0 to 10 according to their personal perceptions of pain and shoulder function. On these scales, a low value represented a low degree of pain or poor shoulder function, whereas a high value indicated a high degree of pain or good shoulder function. Additionally, relevant complications with regard to axillary nerve function were recorded at all follow-up visits.

### Statistics

Results were documented in a case report format and registered in a database. A baseline analysis was performed and figures were generated using IBM SPSS statistics 22 (Statistical Package for the Social Sciences, IBM Corporation, Armonk, NY, USA). After a baseline analysis, a bivariate analysis of the dichotomous and numeric parameters was performed using analysis of variance (ANOVA) and chi-square tests. Next, a secondary analysis of the repetitive measurements for all parameters was performed. For these calculations, only patients with a full dataset were included (*n* = 52). A *p*-value <0.05 was used as the cut-off value for significant differences.

We performed multivariate regression analyses for the Constant and ADL scores to control for confounding factors, including age, gender, and fracture complexity (2-part vs. 3- and 4-part fractures).

## Results

### Baseline data

Of the 114 patients who were eligible for the mid-term follow-up, a total of 71 patients were assessed (62 %) at the mid-term follow-up. Fifteen patients (21 %) were male and 56 (79 %) were female. Fifteen patients had 2-part fractures, 48 had 3-part fractures and 8 had 4-part fractures; thus, the 3- and 4-part fractures were aggregated into one group.

The mean age of these patients was 67 ± 13 years (67 ± 14 for 2-part fractures, ranging from 34 to 81 years; 67 ± 13 for 3-/4-part fractures, ranging from 30 to 83 years, *p* = 0.929). The procedure duration was 67 ± 29 minutes (52 ± 18 for 2-part fractures, 71 ± 30 for 3-/4-part fractures, *p* = 0.023). The length of hospital stay was 9 ± 3 days (9 ± 3 for both 2-part and 3-/4-part fractures, *p* = 0.274). The duration of fluoroscopy during surgery was 2.19 ± 2.00 minutes (2.19 ± 2.00 for 2-part fractures, 1.95 ± 1.12 for 3-/4-part fractures, *p* = 0.668; Table 1).

**Table 1:** Patient demographics and perioperative results

	Age	Duration of procedure	Hospital stay	Fluoroscopy
All patients	67 ± 13	67 ± 29	9 ± 3	2.19 ± 2.00
*n* = 71				
2-part fracture group	67 ± 14	52 ± 18	9 ± 3	2.19 ± 2.00
*n* = 15				
3-/4-part fracture group	67 ± 13	71 ± 30	9 ± 3	1.95 ± 1.12
*n* = 56				
Difference between 2- and 3-/4-part fracture groups	*p* = 0.929	*p* = 0.023	*p* = 0.274	*p* = 0.668

### Complications

Initially, 140 patients were included in our trials. Complications that required revision surgery occurred in 25 of them (18 %). Twelve patients (9 %) sustained a secondary screw perforation in the glenohumeral joint, whereas two of these were caused by primary malpositioning of the implant. In 5 cases (4 %), the humeral head showed secondary head implant loosening. In 3 patients (2 %), the greater tuberosity showed secondary dislocation, 2 patients (1 %) presented with loosening of a single screw, and 1 patient (0.7 %) had a deep wound infection. Two patients (1 %) underwent joint replacement in other hospitals after 2 years.

Of the 71 patients who completed the questionnaire, complications were observed in 9 patients (13 %) including screw perforation (*n* = 7), primary malpositioning of the implant by the surgeon with screw perforation (*n* = 1), and secondary displacement of the greater tuberosity (*n* = 1). All of these complications were observed after 6 weeks and were treated without the implantation of a revision prosthesis. Instead, minor revision surgeries were performed: The screws were readjusted, the position of the implant was corrected and the greater tuberosity was retracted and reattached.

No clinical signs of axillary nerve damage were found during the clinical follow-ups.

### Outcome analysis over time

#### Constant score

**Fig. 3:**
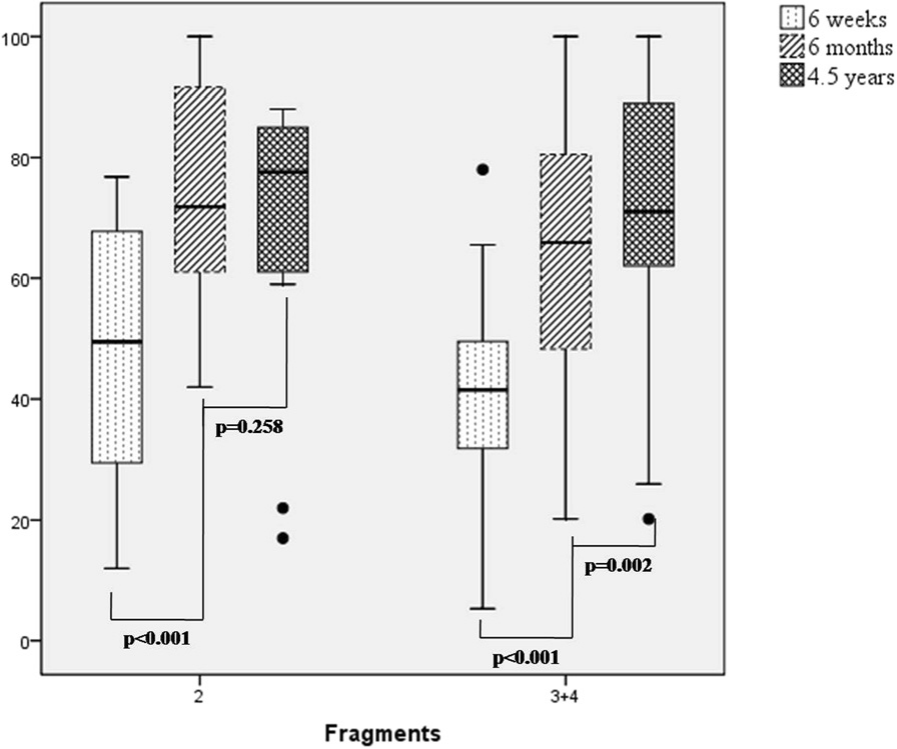
Constant score at follow-up visits for each fracture type

A significant improvement of 21 points was observed between 6 weeks and 6 months for all patients (6 weeks: 35 ± 14, 6 months: 56 ± 18; *p* < 0.001, Fig. 3). Further improvement was observed between 6 months and 4.5 years (4.5 years: 70 ± 21; *p* < 0.001). At the final follow-up, the scores for 2-part fractures were not significantly different from those for complex fractures (2-part fracture: 68 ± 24, 3- and 4-part fracture: 71 ± 20; *p* = 0.660).

#### Activities of daily living score

After an initial increase (6 weeks: 14 ± 5, 6 months: 20 ± 6; *p* < 0.001, Table 2), an improvement in the ADL score was observed at the 4.5-year follow-up (20 ± 8; *p* = 0.049). At the final follow-up, ADL scores remained significantly lower from the pre-trauma values (before trauma: 27 ± 5; *p* < 0.001). The fracture complexity did not influence the final outcome (*p* = 0.719).

**Table 2:** ADL score results from follow-up visits for simple and complex fractures and their corresponding *p*-values

ADL	Before injury	6 weeks	6 months	4.5 years	Before injury – 4.5 years	6 weeks – 6 months	6 months – 4.5 years
All patients	27 ± 5	14 ± 5	20 ± 6	20 ± 8	*p* < 0.001	*p* < 0.001	*p* = 0.049
*n* = 52							
2-part fracture group	29 ± 1	17 ± 7	22 ± 4	21 ± 8	*p* = 0.005	*p* < 0.001	*p* = 0.258
*n* = 12							
3- and 4- part fracture group	27 ± 5	13 ± 4	19 ± 7	20 ± 8	*p* < 0.001	*p* = 0.016	*p* = 0.056
*n* = 40							
Difference between 2- and 3-/4-part fracture groups	*p* = 0.180	*p* = 0.021	*p* = 0.290	*p* = 0.719			

#### VAS score for subjective shoulder function

We observed a significant improvement between 6 weeks and 6 months (6 weeks: 4.9 ± 1.5, 6 months: 7.2 ± 1.8; *p* < 0.001, Table 3) in the VAS score for subjective shoulder function. The results showed a non-significant decrease at the mid-term follow-up (4.5 years: 5.9 ± 2.6; *p* = 0.18). Fracture complexity did not affect the final outcome for this score (*p* = 0.751).

#### VAS pain score

A significant improvement between 6 weeks and 6 months was observed (6 weeks: 3.9 ± 2.0, 6 months: 2.7 ± 1.8; *p* < 0.001, Table 4). A further decrease in pain levels between 6 months and the final follow-up was found for all fracture types (4.5 years: 3.2 ± 3.0; *p* = 0.008). There was no significant difference between 2- and 3-/4-part fractures (*p* = 0.808).

#### Multivariate analysis of data from the final follow-up

A multivariate analysis of the mid-term results showed no influence of gender (95 % CI -14.7 – 8.1, *p* = 0.57, Table 5) or fracture complexity (95 % CI -10.0 – 12.1, *p* = 0.847) on the final Constant score. However, a higher age correlated with a lower score (95 % CI -1.1 – (-0.4), *p* < 0.001).

**Table 3:** VAS scores for subjective function during the follow-up visits for simple and complex fractures and their corresponding *p*-values

Subjective Function	Discharge	6 weeks	6 months	4.5 years	Discharge – 6 weeks	6 weeks – 6 months	6 months – 4.5 years
All patients	3.4 ± 1.7	4.9 ± 1.5	7.2 ± 1.8	5.9 ± 2.6	*p* = 0.003	*p* < 0.001	*p* = 0.176
*n* = 52							
2-part fracture group	4.6 ± 2.2	5.6 ± 1.6	7.5 ± 1.6	6.1 ± 2.9	*p* = 0.376	*p* = 0.044	*p* = 0.951
*n* = 12							
3- and 4- part fracture group	3.1 ± 1.4	4.7 ± 1.4	7.1 ± 1.9	5.8 ± 2.6	*p* = 0.012	*p* = 0.002	*p* = 0.242
*n* = 40							
Difference between 2- and 3-/4-part fracture groups	*p* = 0.007	*p* = 0.053	*p* = 0.459	*p* = 0.751

**Table 4:** VAS score for pain during follow-up visits for simple and complex fractures and their corresponding *p*-values

Pain	Discharge	6 weeks	6 months	4.5 years	Discharge – 6 weeks	6 weeks – 6 months	6 months – 4.5 years
All patients	5.1 ± 3.6	3.9 ± 2.0	2.7 ± 1.8	3.2 ± 3.0	*p* = 0.747	*p* < 0.001	*p* = 0.008
*n* = 52							
2-part fracture group	4.5 ± 1.5	3.5 ± 2.3	2.8 ± 2.0	3.4 ± 3.5	*p* = 0.590	*p* = 0.126	*p* = 0.296
*n* = 12							
3- and 4- part fracture group	5.3 ± 4.0	4.0 ± 1.9	2.7 ± 1.8	3.2 ± 2.9	*p* = 0.575	*p* < 0.001	*p* = 0.017
*n* = 40							
Difference between 2- and 3-/4-part fracture groups	*p* = 0.486	*p* = 0.494	*p* = 0.832	*p* = 0.808

**Table 5:** Multiple regression analysis of factors influencing the final Constant score and ADL outcome

Constant score	B	β	95 % CI of B	*p* value
Gender	-3.26	-0.58	-14.66; 8.13	*p* = 0.568
Fracture complexity	1.07	0.02	-10.01; 12.14	*p* = 0.847
Age	-0.78	-0.53	-1.14; -0.41	*p* < 0.001
ADL	B	β	95 % CI of B	*p* value
Gender	-0.27	-0.02	-5.21; 4.66	*p* = 0.912
Fracture complexity	-0.88	0.05	-5.68; 3.91	*p* = 0.713
Age	-0.28	-0.46	-0.44; -0.12	*p* < 0.001

B = unstandardized regression coefficient, β = standardized regression coefficient

A higher age also correlated with a lower ADL score (95 % CI -0.3 – 0.1, *p* < 0.001), whereas gender (95 % CI -0.3 – 2.5, *p* = 0.912) and fracture complexity (95 % CI -0.8 – 2.4, *p* = 0.713) did not influence the ADL score at the final follow-up.

#### Factors influencing trends in patient outcomes

We compared patients who withstood deterioration in either subjective function or pain between 6 months and mid-term follow-up with patients whose outcome parameters were constant or improved over time. Neither patient group varied with respect to gender, age, fracture complexity, number of complications, or ADL score before trauma (p > 0.05 for each parameter).

### Impact of complications on patient outcomes

Patients with complications did not yield a lower Constant score after 4.5 years (complications: 59 ± 25; no complications: 69 ± 20; *p* = 0.097). Additionally, no differences were observed in their ADL score (complications: 15.88 ± 7.41; no complications: 19.40 ± 8.13; *p* = 0.124), pain (complications: 4.38 ± 3.10; no complications: 3.24 ± 2.59; *p* = 0.143), or subjective shoulder function (complications: 4.66 ± 2.64; no complications: 5.63 ± 2.75; *p* = 0.213).

### Patients who were lost to mid-term follow-up

The patients who could not be contacted did not have a significantly higher age (follow-up: 66 ± 12, lost to follow-up 67 ± 15; *p* = 0.577), more complex fractures (follow-up: 2.86 ± 0.52 fragments, lost to follow-up 2.87 ± 0.62 fragments; *p* = 0.914), or more complications (follow-up: 0.15 ± 0.36, lost to follow-up 0.20 ± 0.41; *p* = 0.513) compared with patients who completed the mid-term follow-up.

## Discussion

In this prospective study, the mid-term results after internal fixation of displaced proximal humeral fractures with a polyaxial locking plate using a less-invasive deltoid split approach are reported.

Our study yielded three main results:

First, there was a significant improvement in the Constant score between 6 months and 4.5 years after surgery (Fig. 3). This improvement may encourage patients to continue working on improving their shoulder function, even after an extended period of time. Röderer et al studied a group of 54 patients with a follow-up of 17 months, an average age of 70 years, and a mean Constant score (without normalization) of 66.8 points. These data are comparable to our results [9]. Additionally, Wu et al conducted a study with a minimum follow-up of 24 months using the locking proximal humerus plate (LPHP). Their data showed slightly better results in the functional outcome; however, their cohort was younger than ours (58.6 ± 11.0 vs. 67 ± 14 years; *n* = 28 vs. *n* = 140) [10]. Ockert et al showed that a good functional outcome is possible at mid-term follow-up (Constant score: 88.4, 95 % CI, 81.7-95.1). However, their patient group was younger than ours (median age 58.2 years), and our multivariate analysis showed that age had a major influence on the final outcome (Table 3). The comparability across studies is relatively low because different implants and surgical approaches were used [11].

Second, complications whereby the implant could be maintained did not alter the final outcome results. This point is of interest because complications are often observed following surgery, particularly in older patients [7, 12]. Given the overall complication rate of 18 % including humeral head replacements, our results corresponded to the literature [13, 14]. The most frequent complication was postoperative screw perforation through the humeral head. This complication could have been caused by the older age of our patients and the high percentage of females; thus, reduced bone quality is suspected as the primary cause. However, a recent publication by Kralinger et al showed that the association between low bone quality and high complication rates is not a definitive factor related to mechanical failure [15]. The comparability to other studies is low because we only included patients with complications that could be treated without the need for joint replacement.

Our third result showed that the ADL score did not reach pre-fracture levels by 4.5 years after injury (Table 2). Unfortunately, this result cannot be compared with the work of Röderer and Wu because the ADL score was not assessed in their study [9, 10]. A study by Hepp et al showed comparable results for the ADL score at the 6-month follow-up. No significant difference was observed between 3 and 6 months in their study; however, our data revealed a significant change between 6 weeks and 6 months (Table 2) [13]. Interestingly, both subjective pain and function were slightly decreased at the last follow-up. This result could be explained by the lack of improvement between 6 months and 4.5 years, which may have led to patient frustration and disappointment. However, patients who showed deterioration were not significantly different in gender, age, fracture morphology, ADL score before trauma, or complications compared with patients who improved. Thus, a lower motivation for physiotherapy or an unhealthy lifestyle could explain the deterioration; however, this possibility was not examined in our study. Clement et al showed that a low activity level before trauma is a decisive risk factor for poor functional outcome after surgery in patients with proximal humeral fractures [16].

Our study has some limitations. To gain as much information as possible, we did not perform a clinical analysis because most of the patients were not able or willing to present for follow-up 4.5 years post-trauma. Therefore, the final follow-up results are based on a questionnaire. We aimed to maintain the response rate as high as possible with this method. However, a clinical follow-up would have enabled a better assessment of clinical complications at the final follow-up. Nevertheless, revision surgery on the shoulder in another clinic was an exclusion from the questionnaire.

Unfortunately, one-third of our patients could not be reached after 4.5 years. It could be assumed that patients with poorer outcomes did not answer the questionnaire, which introduces a potential bias in our study results. To limit this bias, we compared the characteristics of patients who were lost to follow-up with patients who completed the study. We did not find a significant difference between these groups.

Only patients with clinically relevant complaints received a shoulder x-ray according to the local board of ethics. Thus, relevant mid-term complications, such as humeral head necrosis, malunion and nonunion may not have been detected [17]. This is a major issue because patients may have clinically inapparent humeral head necrosis.

Furthermore, several confounders are noteworthy. For example, the patients in our cohort aged during the duration of our trial. Particularly in geriatric patients, this may represent a significant factor. Additionally, the influence of other diagnoses was not analyzed. Osteoporosis, as one example, may significantly cause deterioration in the functional outcome.

## Conclusion

Although internal fixation in proximal humeral fractures using a less-invasive surgical procedure is a feasible treatment option with acceptable complications and considerable improvement during the first six months, many patients were not pain-free and did not reach their pre-fracture activity levels even 4.5 years after injury.
